# Impact of experimental type 1 diabetes mellitus on systemic and coagulation vulnerability in mice acutely exposed to diesel exhaust particles

**DOI:** 10.1186/1743-8977-10-14

**Published:** 2013-04-15

**Authors:** Abderrahim Nemmar, Deepa Subramaniyan, Javed Yasin, Badreldin H Ali

**Affiliations:** 1 Departments of Physiology, College of Medicine and Health Sciences, United Arab Emirates University, P.O. Box 17666, Al Ain, Unite Arab Emirates; 2 Departments of Internal Medicine, College of Medicine and Health Sciences, United Arab Emirates University, P.O. Box 17666, Al Ain, United Arab Emirates; 3 Department of Pharmacology and Clinical Pharmacy, College of Medicine & Health Sciences, Sultan Qaboos University, Muscat 123, P O Box 35, Al-Khod, Sultanate of Oman

**Keywords:** Air pollution, Diesel exhaust particles, Streptozotocin, Type 1 diabetes, Thrombosis, Platelet aggregation, Mice

## Abstract

**Background:**

Epidemiological evidence indicates that diabetic patients have increased susceptibility to adverse cardiovascular outcomes related to acute increases in exposures to particulate air pollution. However, mechanisms underlying these effects remain unclear.

**Methods:**

To evaluate the possible mechanisms underlying these actions, we assessed the systemic effects of diesel exhaust particles (DEP) in control mice, and mice with streptozotocin–induced type 1 diabetes. Four weeks following induction of diabetes, the animals were intratracheally instilled (i.t.) with DEP (0.4 mg/kg) or saline, and several cardiovascular endpoints were measured 24 h thereafter.

**Results:**

DEP caused leukocytosis and a significant increase in plasma C-reactive protein and 8-isoprostane concentrations in diabetic mice compared to diabetic mice exposed to saline or non-diabetic mice exposed to DEP. The arterial PO_2_ as well as the number of platelets and the thrombotic occlusion time in pial arterioles assessed *in vivo* were significantly decreased following the i.t. instillation of DEP in diabetic mice compared to diabetic mice exposed to saline or non-diabetic mice exposed to DEP. Both alanine aminotransferase and aspartate transaminase activities, as well as the plasma concentrations of plasminogen activator inhibitor and von Willebrand factor were significantly increased in DEP-exposed diabetic mice compared to diabetic mice exposed to saline or DEP-exposed non-diabetic mice. The *in vitro* addition of DEP (0.25-1 μg/ml) to untreated mouse blood significantly and dose-dependently induced *in vitro* platelet aggregation, and these effects were exacerbated in blood of diabetic mice.

**Conclusion:**

This study has shown that systemic and coagulation events are aggravated by type 1 diabetes in mice, acutely exposed to DEP and has described the possible mechanisms for these actions that may also be relevant to the exacerbation of cardiovascular morbidity accompanying particulate air pollution in diabetic patients.

## Background

Large body of epidemiological studies have suggested a linkage between particulate air pollution and increased cardiovascular morbidity and mortality [1,2]. Associations between particulate matter with a diameter ≤ 2.5 μm (PM_2.5_) and mortality exists even at low concentrations of air pollutants [1,2]. These epidemiological observations have demonstrated that particles not only exert respiratory effects, but also increase cardiovascular morbidity and mortality [1,2].

An important feature of the epidemiological associations between air pollution and morbidity or mortality is that the acute adverse effects of particulate air pollution appear to be most marked in people with underlying cardiovascular disease, or risk factors such as diabetes mellitus. Indeed, several studies have reported that patients with diabetes mellitus have increased susceptibility to adverse cardiovascular outcomes related to acute increases in exposures to air pollution [3-5].

The vast majority of cases of diabetes are categorized into two broad groups. Type 1 diabetes (known as insulin-dependent diabetes or juvenile-onset diabetes) which is caused by a complete deficiency of insulin secretion resulting from a cellular-mediated autoimmune destruction of the β cells of the pancreas. This form represents about 10% of all forms of diagnosed diabetes. Type 2 diabetes which is much more prevalent (90%) is caused by a combination of resistance to insulin action and an inadequate compensatory insulin secretory response [6,7]. It is well known that cardiovascular complications including thrombosis constitute the major cause of morbidity and mortality in both type 1 and type 2 diabetes [7].

While several studies using type 2 animal model of diabetes have been performed to verify whether or not, and to what extent are the cardiovascular effects of exposure to particulate air pollution exaggerated, experimental studies investigating the effect of particles on animal model of type 1 diabetes are very limited. In relation to type 2 model of diabetes, it has been demonstrated that chronic exposure to PM_2.5_ in high-fat-fed nonatherosclerotic C57 mice aggravates insulin resistance by enhancing inflammation in adipose tissue [8]. More recently, it has been demonstrated that long-term exposure to PM_2.5_ causes glucose intolerance, insulin resistance and inflammation [9,10]. However, using type 1 model of diabetes, as far as we are aware, only one study has reported an aggravation in the increase of 8-Oxo-2′-deoxyguanosine (8-OHdG), a marker of oxidative stress, and endothelin-1 in streptozotocin (STZ)-induced type 1 diabetes in rats compared to non-diabetic ones following the exposure to particulate air pollution [11]. There have been no previous experimental studies on the acute effects of particulate air pollution on coagulation in animal model of type 1 diabetes.

We have recently demonstrated that diesel exhaust particle (DEP) equally increased airway resistance and caused lung inflammation in both STZ-diabetic and nondiabetic mice. However, the occurrence of pulmonary oxidative stress and presence of apoptosis were only seen in DEP-exposed diabetic mice, suggesting that diabetes increased susceptibility to particulate air pollution [12]. In the present study, we aimed at quantifying the effects of pulmonary exposure to DEP in mice on cardiovascular parameters including pial arteriolar thrombosis *in vivo*, platelet aggregation *in vitro*, some markers of inflammation, oxidative stress and fibrinolysis in a mouse model of type 1 diabetes.

## Results

### General characteristic of the diabetic-STZ and non-diabetic mice

The mean body weight in diabetic mice (25.0 ± 1.8 g) was significantly (P < 0.001) lower than that of non-diabetic mice (31.6 ± 1.7 g). The mean blood glucose level was significantly (P < 0.001) increased in diabetic mice (557 ± 62.2 mg/dL) compared to that of non-diabetic mice (130.0 ± 6.9 mg/dL).

### Effect of DEP on systemic inflammation and oxidative stress

**Figure 1:**
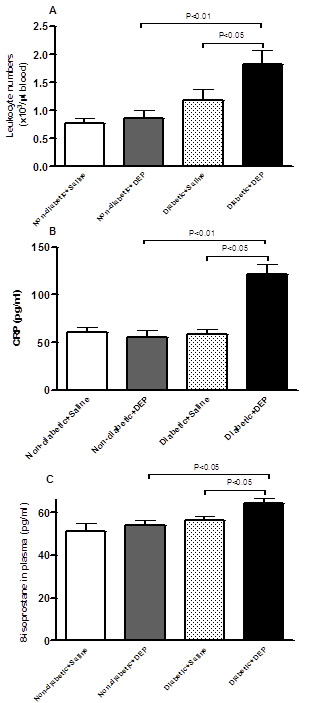
**Effect of diesel exhaust particles (DEP) on leukocyte numbers (A), C-reactive protein (CRP, B) and 8-isoprostane (C) concentrations in plasma.** The latter were measured 24 h following intratracheal instillation of DEP (0.4 mg/kg) or saline in non-diabetic and diabetic mice. Data are mean ± SEM (n = 8).

Figure 1A shows that in diabetic mice, pulmonary exposure to DEP causes leukocytosis. The leukocyte numbers were increased in diabetic mice exposed to DEP compared to diabetic mice exposed to saline or non-diabetic mice exposed to DEP.

The plasma concentration of c-reactive protein (CRP) was significantly increased following DEP exposure in diabetic mice compared to diabetic mice exposed to saline or non-diabetic mice exposed to DEP (Figure 1B).

The concentration of 8-isoprostane, a marker of oxidative stress, was significantly increased after the pulmonary exposure to DEP in diabetic mice versus diabetic mice exposed to saline or non-diabetic mice exposed to DEP (Figure 1C).

### Effect of DEP on arterial PO_2_ and PCO_2_

**Figure 2:**
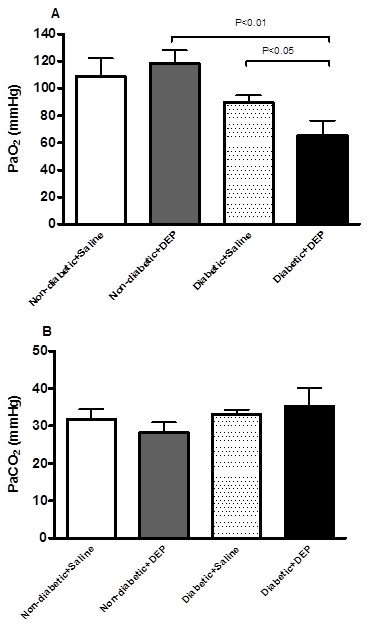
**Effect of diesel exhaust particles (DEP) on arterial PO**_**2 **_**(A) and PCO**_**2 **_**(B).** The latter were measured 24 h following intratracheal instillation of DEP (0.4 mg/kg) or saline in non-diabetic and diabetic mice. Data are mean ± SEM (n = 8).

In non-diabetic mice, i.t. instillation of DEP did not affect the PaO_2_. The decrease in PaO2 was not statistically significant between non-diabetic + saline versus diabetic + saline mice. Interestingly, the PaO_2_ was significantly decreased in diabetic mice exposed to DEP compared to diabetic mice exposed to saline or non-diabetic mice exposed to DEP (Figure 2A). No difference in the PaCO_2_ was found between the 4 different groups (Figure 2B).

### Effect of DEP on alanine aminotransferase (ALT) and aspartate transaminase (AST) activities in plasma

**Figure 3:**
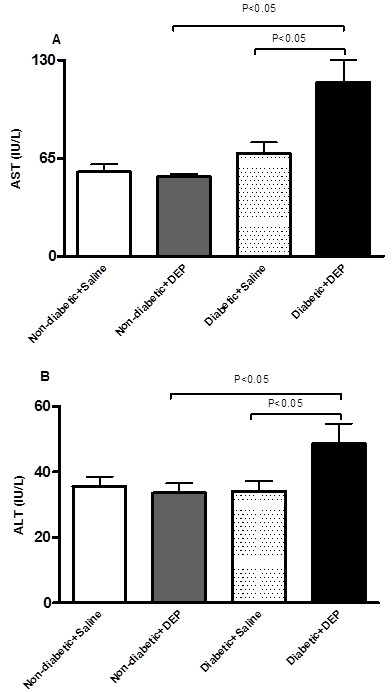
**Effect of diesel exhaust particles (DEP) on aspartate transaminase (AST, A) and alanine aminotransferase (ALT, B) activities in plasma.** The latter were measured 24 h following intratracheal instillation of DEP (0.4 mg/kg) or saline in non-diabetic and diabetic mice. Data are mean ± SEM (n = 8).

In non-diabetic mice, DEP administration did not affect the plasma activities of AST and ALT compared to saline-exposed mice. No difference in the enzyme activities was found between saline-treated diabetic and saline-treated non-diabetic mice. However, the AST and ALT activities were increased in DEP-exposed diabetic mice compared to diabetic mice exposed to saline or DEP-exposed non-diabetic mice (Figure 3).

### Effect of DEP on circulating platelet numbers and photochemically-induced thrombosis in pial arterioles

**Figure 4:**
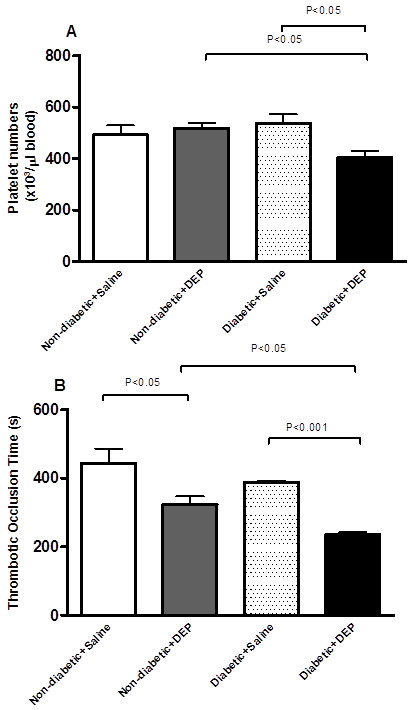
**Effect of diesel exhaust particles (DEP) on circulating platelet numbers (A) and thrombotic occlusion time in pial arterioles (B).** The latter were measured 24 h following intratracheal instillation of DEP (0.4 mg/kg) or saline in non-diabetic and diabetic mice. Data are mean ± SEM (n = 8).

Figure 4A shows that in non-diabetic mice, DEP administration did not affect the number of circulating platelets compared to saline-exposed mice. No difference in platelet numbers was found between saline-treated diabetic and saline-treated non-diabetic mice. In diabetic mice exposed to DEP, the number of platelets was significantly decreased compared to saline-treated diabetic mice or DEP-treated non-diabetic mice.

In non-diabetic mice treated with DEP, the thrombotic occlusion time was significantly shortened compared to saline-treated non-diabetic mice. Similarly, the thrombotic occlusion time was significantly decreased in DEP-treated diabetic mice versus saline-treated diabetic mice. Interestingly, the degree of shortening in the occlusion time was significantly greater in DEP-exposed diabetic mice compared to DEP-exposed non-diabetic mice (Figure 4B).

### Effect of DEP on platelet aggregation in whole blood *in vitro*

**Figure 5:**
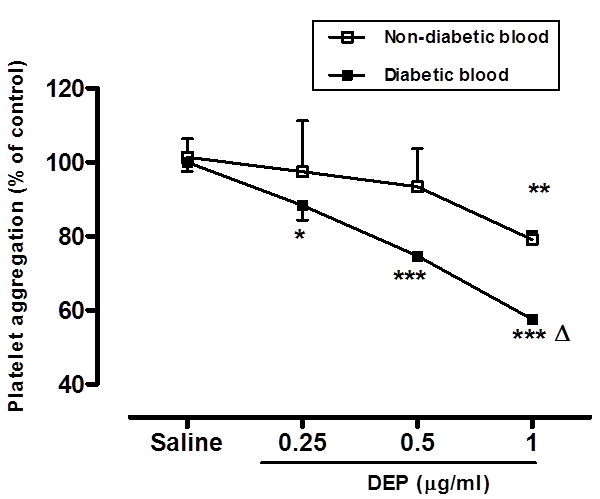
**Direct *in vitro *effect of diesel exhaust particles (DEP) on platelet aggregation in whole blood of untreated non-diabetic and diabetic mice.** Platelet aggregation in untreated non-diabetic and diabetic mouse whole blood 3 min after the addition of saline or DEP (0.25-1 μg/ml) was assessed. The degree of platelet aggregation following DEP exposure was expressed in percent of control (saline-treated diabetic or non-diabetic blood). Data are mean ± SEM (*n* = 3-5). *: P < 0.05 compared with saline-treated blood within the same group. **: P < 0.005 compared with saline-treated blood within the same group. ***: P < 0.0001 compared with saline-treated blood within the same group. ^**^: P < 0.0001 between diabetic and non-diabetic groups for the same given DEP concentration.

Figure 5 illustrates that low concentrations of DEP (0.25–1 μg/ml blood) caused platelet aggregation in a dose-dependent manner. In non-diabetic mouse blood, the effect was significant at concentrations of 1 μg/ml (P < 0.005). In diabetic mouse blood, a clear dose-dependent effect of DEP on platelet aggregation was observed. The effect of DEP on platelet aggregation was significant at 0.25 (P < 0.05), 0.5 (P < 0.0001) and 1 μg/ml (P < 0.0001). Moreover, in diabetic blood, the effect observed at 1 μg/ml was statistically significant (P < 0.0001) compared with the same dose in non-diabetic mouse blood.

### Effect of DEP on plasma concentrations of von Willebrand factor (vWF) and total plasminogen activator inhibitor-1 (PAI-1)

**Figure 6:**
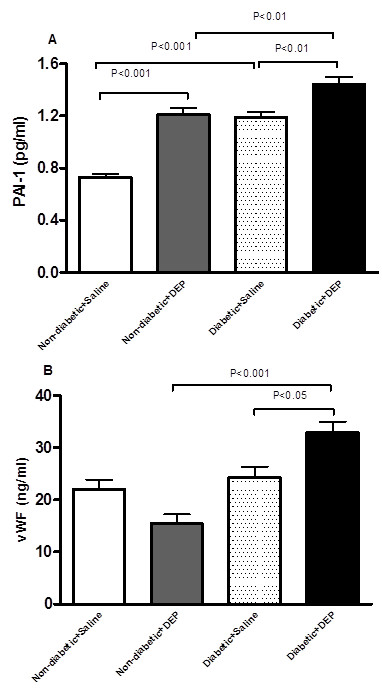
**Effect of diesel exhaust particles (DEP) on plasminogen activator inhibitor 1 (PAI-1, A) and von Willebrand factor (vWF, B) concentrations in plasma.** The latter were measured 24 h following intratracheal instillation of DEP (0.4 mg/kg) or saline in non-diabetic and diabetic mice. Data are mean ± SEM (n = 8).

Figure 6A shows that DEP exposure in both diabetic and non-diabetic mice causes a significant increase of PAI-1 concentration compared to their respective controls. PAI-1 was increased in a greater fashion in the non-diabetic + saline group versus non-diabetic + DEP group (+35%, P < 0.001) compared to diabetic + saline versus diabetic + DEP (+21%, P < 0.01). The increase of PAI-1 in diabetic mice exposed to DEP was significantly higher compared to non-diabetic mice exposed to DEP (+19%, P < 0.01).

The plasma concentration of vWF was not significantly affected following DEP exposure in non-diabetic mice. However, in diabetic mice exposed to DEP, vWF significantly increased compared to saline-exposed diabetic mice or DEP-exposed non-diabetic mice (Figure 6B).

## Discussion

In the present study, we showed an increased cardiovascular vulnerability of diabetic mice to particulate air pollution. We found an aggravation of the impact of acute exposure to DEP in diabetic mice substantiated by increase of systemic inflammation (leukocytosis and CRP), oxidative stress (8-isoprostane), hypoxemia, hepatotoxicity and acceleration of coagulation comprising thrombosis *in vivo*, platelet aggregation *in vitro*, and the increase in plasma concentrations of PAI-1 and vWF.

In the present study, we used a pertinent animal model of type 1 diabetes, i.e. STZ-induced diabetes in mice [11,13] and assessed the acute effects of a relevant type of pollutant particles, namely DEP. The dose of DEP used here 0.4 mg/kg (10 μg/mouse) is lower than the dose previously tested, i.e. 0.5 mg/kg (15 μg/mouse) or 1 mg/kg (30 μg/mouse) because we hypothesized that the effects of DEP would be aggravated in STZ-induced type 1 diabetes in mice. DEP was given to mice by i.t. instillation because it provides more accurate dosing, given that mice are nose breathers that filter most inhaled particles [14]. In 2002, the United States Environmental Protection Agency reported a range of maximal city PM_10_ concentrations between 26 and 534 μg/m^3^[15]. Several large cities in the world have much higher levels of PM_10_, with annual averages of 200 to 600 μg/m^3^ and peak concentrations frequently exceeding 1,000 μg/m^3^[16]. Using the highest value in the United States and assuming a minute ventilation of 6 l/min (~8.6 m^3^ over 24 hours) for a healthy adult at rest, the total dose of PM inhaled over 24 hours would be 4,614 μg [17]. Exposure of a human to a daily dose of 4,614 μg of PM would correspond to more than 35 μg of PM exposure for a mouse (25 grams in size) with minute ventilation of 35–50 ml/min [17]. The dose we tested here (10 μg/mouse) is lower than the comparative human dose of ± 35 μg/mouse reported by Mutlu et al. [17].

Our data show that in non-diabetic mice, at the dose and regimen studied, DEP did not affect the number of leukocytes or the CRP concentration in plasma. Previously, 24 h post-exposure to higher doses of DEP, i.e. 15 μg/mouse (0.5 mg/kg) or 30 μg/mouse (1 mg/kg), we found no increase in the number of leukocytes [18,19]. No significant differences were observed between control diabetic and non-diabetic mice. Interestingly, DEP exposure induced a leukocytosis and a significant increase of CRP, indicating the occurrence of systemic inflammation. Our finding corroborate epidemiological studies that have reported positive associations between air pollution and indicators of systemic inflammation such as leukocyte numbers, interleukin 6 and CRP [3,20]. Remarkably, it has been reported that these associations were stronger and most consistent in individuals with diabetes [3]. We have recently reported that repeated exposure to DEP causes an increase in CRP concentration and that the pre-treatment with the anti-inflammatory and antioxidant curcumin returned the CRP concentrations to control levels [21].

We have recently demonstrated that 24 h following their i.t. instillation, DEP (0.5 and 1 mg/kg) caused pulmonary and systemic oxidative stress responsible for systemic inflammation, and that the pretreatment with a cysteine prodrug L-2-oxothiazolidine-4-carboxylic acid abrogated these effects through its ability to balance oxidant-antioxidant status [18]. In the present study, as a marker for oxidative stress, we selected to measure the plasma concentrations of 8-isoprostane. Isoprostanes are a family of eicosanoids of nonenzymatic origin, produced by the random oxidation of tissue phospholipids by oxygen radicals. Elevated levels of isoprostanes have been found in serum, plasma, and urine of heavy smokers [22] and lung tissue of mice expose to carbon nanoparticles [23]. Here, we found that plasma 8-isoprostane concentrations were significantly increased after the pulmonary exposure to DEP in diabetic mice versus diabetic mice exposed to saline or non-diabetic mice exposed to DEP. It is well-established that oxidative stress plays a key role in the pathogenesis of of diabetes mellitus [24]. Diabetic patients usually have significantly elevated concentrations of 8-OHdG in their serum and decreased levels of glutathione [24]. Our data are in agreement with previous findings which reported that PM_2.5_ exposure causes aggravation of plasma oxidative stress in STZ-diabetic rats compared to nondiabetic rats [11].

While PaCO_2_ was not affected by DEP in both diabetic and non-diabetic mice, the PaO_2_ was significantly decreased in diabetic mice exposed to DEP compared to diabetic mice exposed to saline or non-diabetic mice exposed to DEP. We recently demonstrated that DEP exposure in hypertensive mice significantly decreased the PaO_2_ compared with DEP-treated normotensive mice. Moreover, using a rat model of cisplatin-induced acute renal failure, we have recently shown a decrease in PaO_2_ following DEP exposure [25]. Our findings are in agreement with epidemiological studies that suggested that pollution may result in hypoxemia and that these effects might be most relevant in older and sicker individuals [26,27].

In non-diabetic mice, DEP administration did not affect the plasma activities of AST and ALT compared to saline-exposed mice. No difference in the enzyme activities was found between saline-treated diabetic and saline-treated non-diabetic mice. Remarkably, the AST and ALT activities were increased in DEP-exposed diabetic mice compared to diabetic mice exposed to saline or DEP-exposed non-diabetic mice, indicating that DEP causes tissue damage in diabetic mice. Exposure to PM_2.5_ in healthy mice did not affect AST and ALT activities [28]. However, it has been reported that pulmonary exposure of obese diabetic mice to DEP causes an increase in the activities of AST, ALT, the ratio of liver weight, and the magnitude of fatty change of the liver in histology [29]. Epidemiological and clinical studies are needed to verify the occurrence of liver injury following the exposure to particulate air pollution in susceptible population.

A strong epidemiologic association has been observed between increased levels of PM and hospitalizations for heart disease among those who had diabetes compared with those who did not [5]. The risk of coronary heart disease, stroke, and peripheral arterial disease is increased in persons with diabetes [30]. Several experimental studies have reported that exposure to particles causes prothrombotic effects in the ear vein of rats [31], femoral vein and artery of hamsters [32-35] carotid artery of mice [17] and pial venule or arterioles of mice [18,36]. Our data confirms the occurrence of prothrombotic effects following the exposure to DEP in non-diabetic mice compared to saline-treated non-diabetic mice. Similarly, we found a shortening in the thrombotic occlusion time in diabetic mice exposed to DEP compared to those exposed to saline. Interestingly, the degree of shortening in the thrombotic occlusion time was significantly greater DEP-exposed diabetic mice compared to DEP-exposed non-diabetic mice. Recently, we reported an aggravation of thrombotic events in hypertensive mice [19].

Along with the potentiation of prothrombotic effect in diabetic mice exposed to DEP, we found a significant decrease in platelet numbers in DEP-exposed diabetic mice compared to DEP-exposed non-diabetic mice or saline-exposed diabetic mice, this is indicative of platelet activation *in vivo*. A decrease of platelet numbers following exposure to particles has been reported from experimental and clinical studies [18,37].

It has been suggested that inhaled particles may lead to systemic inflammatory response through the release of inflammatory mediators and oxidative stress within the lungs and/or systemically [1,38]. Additional experiments showed that air pollution exposure is associated with rapid changes in autonomic nervous system balance, favouring sympathetic nervous system activation and parasympathetic withdrawal [1,38]. Other lines of evidence also suggest that nanoparticulate inhalation can rapidly translocate from through the alveolar capillary barrier and directly affect the cardiovascular system [1,38-40]. Because arteriolar thrombosis measured *in vivo* in our model depends mainly on the intensity of the vascular lesion and subsequent platelet recruitment and aggregation, we wanted to test the direct effect of DEP on platelet aggregation in whole blood of diabetic and non-diabetic mice *in vitro*. We, and others, have previously reported that DEP cause platelet aggregation [36,41]. Our *in vitro* observations confirmed the occurrence of platelet aggregation following the addition of DEP. Clearly, an aggravated effect was observed in diabetic mouse blood with dose-dependent and significant graded effects at 0.25, 0.5 and 1 μg/ml DEP. Interestingly, in diabetic blood, the effect observed at 1 μg/ml was statistically significant compared with the same dose in non-diabetic mouse blood. This *in vitro* finding corroborates our *in vivo* observation. Such observation has, as far as we are aware, never been reported before. Our data corroborate a recent human study which reported that PM exposure was associated with a change in platelet function toward a greater prothrombotic tendency in diabetic patients [42].

Exposure to DEP in both diabetic and non-diabetic mice caused a significant increase of PAI-1 concentration compared to their respective controls. However, PAI-1 was increased in a greater fashion in the non-diabetic + saline group versus non-diabetic + DEP (+35%) group compared to diabetic + saline versus diabetic + DEP (+21%) mice. This difference can be explained by the fact that the concentration of PAI-1 in diabetic + saline group was significantly increased compared to non-diabetic + saline group. This finding corroborates the study of Tagher et al. [43] who found that PAI-1 concentration was significantly higher in patient with type 1 diabetes compared to healthy controls. We found a significant increase of circulating PAI-1 in diabetic mice exposed to DEP compared to diabetic mice exposed to saline or non-diabetic mice exposed to DEP. Raised concentrations of circulating PAI-1 have been acknowledged as an independent risk factor for the development of ischemic cardiovascular events [44,45]. The concurrent increase of plasma PAI-1 and decrease of PaO_2_ that we observed corroborate the finding of pinsky et al. [46] who demonstrated that enhanced expression of PAI-1 is an important mechanism suppressing fibrinolysis under conditions of low oxygen tension. We recently reported that repeated exposure to DEP in healthy mice caused an increase of plasma PAI-1 concentration, and another study showed an increase in PAI-1 mRNA and protein concentrations in lung and adipose tissue of mice treated with PM [47]. Moreover, Erdely et al. [48] showed that pulmonary exposure to carbon nanotube increased the active form as well as total PAI-1 in the circulation. We also found an increase of vWF in DEP-treated diabetic mice compared to saline-treated diabetic mice or DEP-treated non-diabetic mice. vWF reflects endothelial cell release and probably vascular reactivity. Vascular reactivity could results from the oxidative stress or direct effects of DEP that have presumably translocated into the systemic circulation. Moreover, vWF can mediate platelet adhesion to damaged endothelium, this could explain at least partly the observed exaggerated prothrombotic effects of DEP in diabetic mice. Elevated levels of vWF were observed in association with increased concentrations of particulate matter in patients with coronary heart disease [49]. In healthy mice, increased vWF expression on hepatic endothelium was detected after intraarterial administration of nanoparticles [50].

Collectively, our data show an aggravation of various systemic and coagulation endpoints *in vivo* and *in vitro* in diabetic mice acutely exposed to DEP compared to non-diabetic mice exposed to DEP or diabetic mice exposed to saline. These exacerbations could be ascribed to the increase of systemic oxidative stress and inflammation observed particularly in diabetic mice exposed to DEP (Figure 1). Indeed, both oxidative stress and inflammation were reported to play a critical role in the cardiovascular effects of particulate air pollution [1,18,36] and diabetes [51]. Nevertheless, further studies are required clarify the mechanisms underlying the effect of type 1 diabetes and DEP on the cardiovascular system and whether the observed effects are strain-dependent. A murine strain differences in airway inflammation caused by DEP has been previously reported [52].

We conclude that systemic and coagulation events are aggravated in type 1 diabetic mice acutely exposed to DEP. Our findings provide possible plausible explanation for the exacerbation of cardiovascular morbidity accompanying particulate air pollution in diabetic patients. Additional experiments are needed to evaluate the chronic effect of DEP on type 1 diabetes and determine whether the observed effects are related to the DEP-associated components or by particles themselves.

## Material and methods

### DEP

The DEP (SRM 2975) were obtained from the National Institute of Standards and Technology (NIST, Gaithersburg, MD, USA), and were suspended in sterile saline (NaCl 0.9%) containing Tween 80 (0.01%). To minimize aggregation, particle suspensions were always sonicated (Clifton Ultrasonic Bath, Clifton, New Jersey, USA) for 15 min and vortexed before their dilution and prior to intratracheal (i.t.) administration. Control animals received saline containing Tween 80 (0.01%).

DEP suspension that we used here has been previously analyzed by transmission electron microscopy [53,54]. This has revealed the presence numerous small aggregates of carbonaceous particles, and substantial amount of ultrafine (nano)-sized particle (less than 100 nm) aggregates were seen. Most of the observed aggregates were <1 μm in the largest diameter [53,54].

The endotoxin concentration in the DEP solution and saline used was quantified, as described by the manufacturer, by chromogenic Limulus Amebocyte Lysate (Pierce, Rockford, IL) test. The concentration was lower than the detection limit (0.1 EU/ml) in the saline and DEP solutions.

### Animals and treatments

This project was reviewed and approved by the Institutional Review Board of the United Arab Emirates University, College of Medicine and Health Sciences, and experiments were performed in accordance with protocols approved by the Institutional Animal Care and Research Advisory Committee.

Male TO mice (HsdOla:TO, Harlan, UK) were housed in light (12-h light:12-h dark cycle) and temperature-controlled (22 ± 1°C) rooms. They had free access to commercial laboratory chow and were provided tap water ad libitum.

Type 1 diabetes mellitus was induced in male TO mice (6 to 8 weeks old) by intraperitoneal (i.p.) injection of 200 mg/kg body weigh STZ (Sigma Chemical, St. Louis, MO) [55,56]. Tail vein blood glucose samples were measured before and during 4 weeks after injection to ensure induction of diabetes. The non-diabetic (control) mice were injected i.p. with the vehicle (0.1 mol/l citrate buffer, pH 4.5). Four-weeks post-STZ injection, mice were anesthetized with sodium pentobarbital (60 mg/kg, i.p.), placed supine with extended neck on an angled board. A Becton Dickinson 24 Gauge cannula was inserted via the mouth into the trachea. The DEP suspensions (0.4 mg/kg) or saline-only were instilled intratrachealy (i.t.) (50 μl) via a sterile syringe and followed by an air bolus of 50 μl to diabetic or non-diabetic mice.

### Blood collection and analysis

24 h after the the i.t. administration of either saline or DEP, the animals were anesthetized, as described above, and blood was drawn from the inferior vena cava in EDTA (4%). A sample was used for platelets and white blood cells counting using an ABX VET ABC Hematology Analyzer with a mouse card (ABX Diagnostics, Montpellier, France). The remaining blood was centrifuged at 4°C for 15 min at 900 *g* and the plasma samples were stored at −80°C until further analysis.

### Determination of CRP, 8-isoprostane, ALT, AST, vWF and total PAI-1 levels in plasma

The concentrations of CRP (Uscn Life Science Inc, Wuhan, China), 8-isoprostane (Cayman Chemicals, Michigan, USA), PAI-1 (Molecular Innovation, Southfield, MI, USA) and vWF (Uscn Life Science Inc, Wuhan, China) were determined using ELISA Kits. The activities of AST and ALT were measured using standard laboratory methods with LX20 multiple automated analyser (Beckman Coulter, CA, USA).

### Arterial PO_2_ and PCO_2_ analysis

Arterial blood gases were measured in separate animals following the protocol described above. Immediately after the anesthesia, arterial blood was obtained via the abdominal aorta with a heparinized 24-gauge needle. Analysis was performed immediately after collection with an OPTI CCA-TS blood gas analyzer (OPTI Medical, Roswell, GA, USA).

### Experimental pial cerebral arterioles thrombosis model

In a separate experiment, *in vivo* pial arteriolar thrombogenesis was assessed 24 hours after the i.t. instillation of either DEP or saline, according to a previously described technique [18,36]. Briefly, the trachea was intubated after induction of anesthesia with urethane (1 mg/g body weight, i.p.), and a 2 F venous catheter (Portex, Hythe, UK) was inserted in the right jugular vein for the administration of fluorescein (Sigma, St. Louis, MO, USA). After that, a craniotomy was first performed on the left side, using a microdrill, and the dura was stripped open. Only untraumatized preparations were used, and those showing trauma to either microvessels or underlying brain tissue were discarded. The animals were then placed on the stage of a fluorescence microscope (Olympus, Melville, NY, USA) attached to a camera and DVD recorder. A heating mat was placed under the mice and body temperature was raised to 37°C, as monitored by a rectal thermoprobe connected to a temperature reader (Physitemp Instruments, NJ, USA). The cranial preparation was moistened continuously with artificial cerebrospinal fluid of the following composition (mM): NaCl 124, KCl 5, NaH_2_PO_4_ 3, CaCl_2_ 2.5, MgSO_4_.4, NaHCO_3_ 23 and glucose 10, pH 7.3-7.4. A field containing arterioles 15–20 μm in diameter was chosen. Such a field was taped prior to and during the photochemical insult, which was carried out by injecting fluorescein (0.1 ml/mouse of 5% solution) via the jugular vein, which was allowed to circulate for 30–40 sec. The cranial preparation was then exposed to stabilized mercury light. The combination produces endothelium injury of the arterioles. This, in turn, causes platelets to adhere at the site of endothelial damage and then aggregate. Platelet aggregates and thrombus formation grow in size until complete vascular occlusion. The time from the photochemical injury until full vascular occlusion (time to flow stop) in arterioles were measured in seconds. At the end of the experiments, the animals were euthanized by an overdose of urethane.

### Platelet aggregation in mouse whole blood

The platelet aggregation assay in whole blood was performed, with slight modification, as described before [57]. After anesthesia, blood from untreated diabetic and non-diabetic mice was withdrawn from the inferior vena cava and placed in citrate (3.2%), and 100-μl aliquots were added to the well of a Merlin coagulometer (MC 1 VET; Merlin, Lemgo, Germany). The blood samples were incubated at 37.2°C with either saline (control) or DEP (0.25-1 μg/ml) for 3 min, and then stirred for another 3 min. At the end of this period, 25-μl samples were removed and fixed on ice in 225 ml cellFix (Becton Dickinson). After fixation, single platelets were counted in a VET ABX Micros with mouse card (ABX, Montpellier, France). The degree of platelet aggregation following DEP exposure was expressed as a ℅ of control (saline-treated blood).

### Statistics

Data were expressed as means ± SEM, and were analyzed with GraphPad Prism Version 4.01 for Windows software (Graphpad Software Inc., San Diego, USA). For the *in vivo* results, comparisons between the four groups were performed by analysis of variance ANOVA, followed by Newman-Keuls multiple-range tests. For the *in vitro* platelet aggregation data, comparison was performed using unpaired student’s t-test. P values <0.05 are considered significant.

## Competing interests

The authors declare that they have no competing financial interests.

## Authors’ contributions

AN designed, planned, supervised all the experiments and wrote the article. DS and JY preformed the experiments. BHA contributed in the design of the study and wrote the article. All authors have read and approved the manuscript.
